# The genus *Anthia* Weber in the Republic of South Africa, Identification, distribution, biogeography, and behavior (Coleoptera, Carabidae)

**DOI:** 10.3897/zookeys.143.2075

**Published:** 2011-11-01

**Authors:** Jonathan R. Mawdsley, Terry L. Erwin, Hendrik Sithole, James L. Mawdsley, Alice S. Mawdsley

**Affiliations:** 1 Department of Entomology, MRC 187, National Museum of Natural History, Smithsonian Institution, P. O. Box 37012, Washington, DC 20013-7012 USA; 2 Invertebrates, South African National Parks, P. O. Box 110040, Hadison Park, Kimberley, 8306 South Africa; 3 Cleveland State University, 2121 Euclid Avenue, Cleveland, OH 44114 USA

**Keywords:** *Anthia*, Carabidae, taxonomy, identification, savanna, South Africa, *Apristis promontorii* Péringuey

## Abstract

A key is presented for the identification of the four species of *Anthia* Weber (Coleoptera: Carabidae) recorded from the Republic of South Africa: *Anthia cinctipennis* Lequien, *Anthia circumscripta* Klug, *Anthia maxillosa* (Fabricius), and *Anthia thoracica* (Thunberg). For each of these species, illustrations are provided of adult beetles of both sexes as well as illustrations of male reproductive structures, morphological redescriptions, discussions of morphological variation, annual activity histograms, and maps of occurrence localities in the Republic of South Africa. Maps of occurrence localities for these species are compared against ecoregional and vegetation maps of southern Africa; each species of *Anthia* shows a different pattern of occupancy across the suite of ecoregions and vegetation types in the Republic of South Africa. Information about predatory and foraging behaviors, Müllerian mimicry, and small-scale vegetation community associations is presented for *Anthia thoracica* based on field and laboratory studies in Kruger National Park, South Africa.

## Introduction

**Figure 1.**
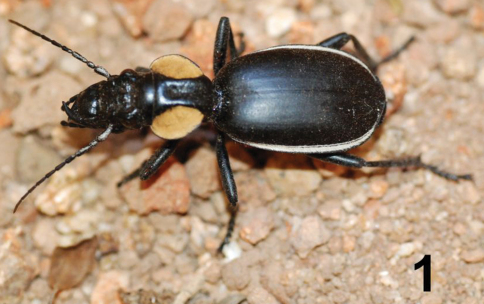
Adult female of *Anthia thoracica* (Thunberg), photographed in the N’waswitshaka Research Camp, Skukuza, Kruger National Park, Republic of South Africa.

**Figure 2.**
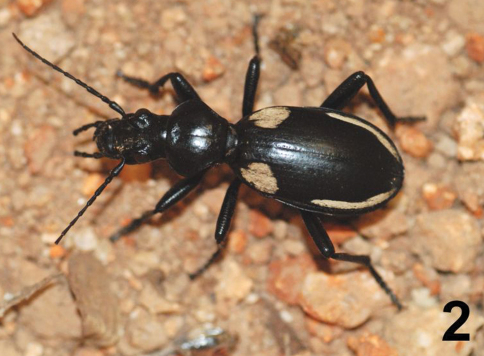
Adult male of *Termophilum homoplatum* (Lequien), photographed in the N’waswitshaka Research Camp, Skukuza, Kruger National Park, Republic of South Africa.

Beetles in the genus *Anthia* Weber are some of the largest and most conspicuous representatives of the family Carabidae in sub-Saharan Africa (Scholtz and Holm 1985; Picker, Griffiths and Weaving 2002). Adults of *Anthia* species (Figure 1) and those of the closely related genus *Termophilum* Basilewsky (Figure 2) are boldly patterned in black and white or yellow stripes and/or spots. These beetles are armed with potent chemical defenses and are able to spray highly concentrated acidic secretions over a distance of a meter or more, often directed at the head and eyes of an attacker (Scott et al. 1975; Huey and Pianka 1977). Sympatric species of *Anthia* and *Termophilum* often have similar color patterns, a fact that has been interpreted as a possible example of Müllerian mimicry (Marshall and Poulton 1902; Huey and Pianka 1977). The genus *Anthia* and its relatives offer remarkable opportunities for studies of chemical ecology, as well as aposematic color patterns and the evolution of mimetic coloration.

Unfortunately, studies of the ecology, evolution, and behavior of these beetles have long been hampered by the lack of reliable, illustrated identification materials for species of *Anthia* and related genera. The two older revisions by Péringuey (1896) and Obst (1901), and the catalogue of species by Csiki (1929) often conflict in the placement of individual taxa, and these historic works generally lack good habitus images or line drawings that could be used by non-specialists to identify species in this group. Fortunately, recent revisionary work by (Schmidt (2001, 2002) and Schmidt and Gruschwitz (2002) has helped to clear up many longstanding nomenclatural issues in this group and provide a firm foundation for the development of reliable identification materials.

The present treatment was written to provide identification materials for species of *Anthia* (*sensu* Basilewsky 1950) from the present-day Republic of South Africa (RSA). This paper forms part of a series of studies on southern African Carabidae and Cicindelidae, with a particular focus on taxa associated with the Kruger National Park in the Republic of South Africa. Previous contributions in this series include (Mawdsley and Sithole (2008, 2009), (Mawdsley (2009, 2011), and Mawdsley et al. (2011). The goal of this series of publications is to provide high-quality identification materials for groups of ground beetles and tiger beetles which are of potential interest to conservation biologists, environmental scientists, and park and natural area managers.

Unlike many parts of sub-Saharan Africa, the Republic of South Africa has been well sampled for *Anthia* and related genera and there is a wealth of museum material available for study. Even with this wealth of material, there has been considerable confusion in the literature regarding the identification and appropriate names to assign to these species. At the level of generic names, many authors follow Péringuey (1896), Obst (1901), and Csiki (1929) in applying the genus name *Anthia* to a larger group of approximately 66 species from sub-Saharan Africa, southern Asia, and India. In this paper, we follow Basilewsky (1950), (Schmidt (2001, 2002), Schmidt and Gruschwitz (2002), and (Lorenz (2005a, 2005b) in restricting the use of the genus name *Anthia* Weber to the group of approximately fourteen species exhibiting sexual dimorphism in the structure of the pronotum and/or mandibles.

There has also been considerable confusion regarding the number of species of *Anthia* in southern Africa. Csiki (1929) represents the most conservative estimate, recognizing just two species, *Anthia maxillosa* (which he called *Anthia fabricii*) and *Anthia thoracica*. Péringuey (1896) recognized an additional five species for a total of seven. In this paper, we recognize four species that can be readily separated on the basis of attributes of pronotal and elytral vestiture and surface sculpture, as well as by the structure of the male genitalia. Our conclusions parallel those of (Schmidt (2001, 2002) and Schmidt and Gruschwitz (2002) as well as the late Pierre Basilewsky, who studied this genus for many years. According to Basilewsky’s identification labels on museum specimens that we examined, Basilewsky recognized the same four species from South Africa that we recognize here.

## Materials and methods

We examined specimens of species of *Anthia*, *Termophilum*, and allied genera in the collections of the following museums: Field Museum of Natural History, Chicago, Illinois (FMNH); Kruger National Park Museum (Scientific Services), Skukuza, South Africa (KNPC); South African National Collection of Insects, Pretoria, South Africa (SANC); National Museum of Natural History, Smithsonian Institution, Washington, D.C. (NMNH); Transvaal Museum, Pretoria, South Africa (TMSA).

Field observations on adults of *Anthia thoracica* and other Anthiini were conducted during month-long visits by the senior author to the Kruger National Park in 2007, 2008, 2009, and 2010. Systematic field surveys for Anthiini and other diurnally active Carabidae were conducted in areas where adults of Anthiini had been collected historically in the park, or where adult beetles had been observed recently by park staff. Our surveys focused primarily on the Skukuza Ranger District in the central portion of the park, with field trips north to Satara, Letaba, Olifants, and Shingwedzi and south to Pretoriuskop. In surveying these areas we employed a variety of techniques, including driving surveys, walking surveys, and pitfall trapping (Mawdsley et al. 2011). Driving surveys were quickly identified as the most productive of these survey techniques for large Anthiini and consequently were widely applied throughout the southern area of Kruger National Park. In this survey approach, a party of four searchers drove slowly in a car along secondary sand or gravel roads and stopped whenever large carabid beetles were observed running on or across the road, or in vegetation along the side of the road. When a beetle was observed, the car was stopped and one or more persons left the vehicle in order to capture the beetle. In walking surveys, a group of four searchers walked slowly along segments of sand and gravel roads, looking for Carabidae running among dead leaves, vegetation, or on bare ground. We also deployed pitfall traps of different diameters in areas where Anthiini were observed, although we found through experience that our small-diameter traps were less productive at capturing large-bodied Carabidae and were frequently raided by baboons, mongoose, and other predatory vertebrates. We conducted field surveys under all weather conditions, from sunny days with no cloud cover, to sunny days with afternoon thunder showers, to overcast, rainy days. Ambient air temperatures during surveys varied from 18C to 35C.

The driving technique proved to be a particularly productive method for collecting Anthiini, as well as other large Carabidae such as species of *Tefflus* Leach (Mawdsley et al. 2011). In addition to adults of *Anthia thoracica*, this method resulted in capture of adults of *Termophilum burchelli* (Hope), *Termophilum homoplatum* (Lequien), *Termophilum massilicatum* (Guérin), and *Cypholoba graphipteroides* (Guérin). The driving technique has the advantage of being able to cover a large geographic area within a relatively limited amount of time. The disadvantage to this technique is that many smaller-bodied species of Carabidae and Cicindelidae are overlooked, particularly those that are cryptically colored. However, we found that these taxa (particularly Carabidae of the genus *Graphipterus* Latreille and Cicindelidae of the genera *Dromica* Dejean and *Lophyra* Motschulsky) were easily detected during walking surveys.

For studies of beetle biology in captivity, individual adult beetles were captured by hand and placed singly into large 4-liter plastic holding containers containing a shallow layer of sand and gravel in the bottom. Each container was provided with a small ball of cotton soaked in water, to provide a water source for the adult beetles. To examine prey preferences, we provided each captive beetle with a variety of potential food items, primarily insects and other arthropods which were collected at lights at night in the Skukuza research camp. We recorded acceptance/rejection of each potential food item and the associated order and family of each prey item offered to the beetles.

Voucher specimens of beetles collected in Kruger National Park are deposited in the KNPC, NMNH, and TMSA collections.

Attributes of the abdominal ventral sterna are referred to using the numbering system generally accepted in Carabid studies, i.e., the sternum divided medially by the hind coxae is sternum II (the first being hidden) and the last visible is sternum VII (Liu et al. 2011).

### Genus Anthia

Weber, 1801

http://species-id.net/wiki/Anthia

Anthia Weber (1801:17).
Carabus sexguttatus Fabricius (1775:236); subsequent designation by Latreille (1810:426). Type Species.
Pachymorpha Hope (1838:51); synonymized by Basilewsky (1950:80).
Thoracolobus Gistel (1857:50); synonymized by Csiki (1929:377).

#### Diagnosis.

Body large and massive, adults of South African species always 40 mm or greater in length; body black or dark brown, usually with yellow or white setae and pubescence. Prothorax cordiform, distinctly expanded laterally and usually with large lateral flanges. Mandibles and prothorax sexually dimorphic: mandibles elongate in males, shorter in females; base of pronotum with two posterior flanges or flattened extensions in males, tumescent without extensions in females. Elytra smooth with rows of minute punctures or feebly striatiopunctate, never markedly striatiopunctate in South African species (although other species in the genus do have striatiopunctate elytra).

#### Recognition from sympatric genera.

Specimens of southern African species of *Anthia* may be readily distinguished from those of allied genera by the presence of broad lateral flanges on the pronotum and the sexual dimorphism in the structure of the mandibles and pronotal base. Most other South African Anthiini also have the elytra markedly striatiopunctate, at least in part. The only sympatric genus with which species of *Anthia* might be confused is *Termophilum* Basilewsky, that contains several large species that are similar in overall appearance and markings with those of the genus *Anthia*. However, species of *Termophilum* have a much simpler pronotal structure that lacks the large lateral flanges and secondary sexual characteristics present in *Anthia* species. Species of *Termophilum* also lack the sexual dimorphism in the mandibles that is seen in species of *Anthia*.

#### Notes on Taxonomy.

Basilewsky (1950) was the first to point out that the generic names *Anthia* Weber and *Pachymorpha* Hope have the same type species, *Carabus sexguttatus* Fabricius. The name *Anthia* clearly has priority over *Pachymorpha*. Basilewsky (1950) noted that a replacement name for *Pachymorpha* was not necessary, as the species formerly classified in that genus fit readily within his restricted concept of the genus *Anthia*, a generic concept which we follow here. *Thoracolobus* was proposed by Gistel (1857) for the two species *Anthia maxillosa* (F.) and *Anthia thoracica* (Thunberg) and is clearly synonymous with *Anthia* Weber as treated here.

#### Key to South African species of *Anthia* Weber 1801

**Table:** 

1	Elytra with a distinct band of white setae along lateral margins	2
–	Elytra lacking distinct band of white setae along lateral margins	*Anthia maxillosa* (Fabricius)
2	Pronotum with lateral patches of white, yellow, or brown setae; aedeagus stout, thick	3
–	Pronotum lacking lateral patches of white, yellow, or brown setae; aedeagus narrow, elongate	*Anthia cinctipennis* Lequien
3	Lateral flanges of pronotum with large patches of dense yellow or brown reclinate seta forming two large round or ovate “spots;” aedeagus stout and thick along entire length (Figure 28)	*Anthia thoracica* (Thunberg)
–	Lateral flanges of pronotum with more-or-less distinct patches of suberect white setae; aedeagus thinner towards base (Figure 31)	*Anthia circumscripta* Klug

### Anthia thoracica

(Thunberg, 1784)

http://species-id.net/wiki/Anthia_thoracica

Figures 1 3–11 28 32 36

Carabus thoracicus Thunberg (1784:69).
Carabus fimbriatus Thunberg (1784:70); synonymized by Dejean (1825:340).
Anthia portentosa Dohrn (1882:246); synonymized by Obst (1901:285).
Anthia thoracica var. *stigmodera* Péringuey (1896:375); synonymized by Csiki (1929:379).
Anthia dohrni Rousseau (1905:8); synonymized by Csiki (1929:379).

#### Type Locality.

“Capite bonae spei” (= Cape of Good Hope).

#### Type Depository.

*Carabus thoracicus* and *Carabus fimbriatus*, Uppsala University, Museum of Evolution, Zoology Section; *Anthia portentosa*, formerly in the Museum für Naturkunde Stettin, and apparently lost in World War II; *Anthia thoracica* var. *stigmodera*, South African Museum, Iziko Museums of Cape Town.

#### Diagnosis.

Easily separated from sympatric species of *Anthia* by the large round or ovate patches of yellow or brown setae on the lateral flanges of the pronotum. *Anthia thoracica* is the most widespread species of *Anthia* in South Africa and although adults are usually encountered singly, the species can be locally abundant.

#### Description.

Body size massive, length of male (exclusive of mandibles) 46.8-52.8 mm, length of female 40.5-50.3 mm. Integument black.

Head elongate, prognathous. Mandibles elongate and sickle-shaped in male, short and stout in female. Male mandibles asymmetrical, with left mandible more markedly recurved than right. Length of right mandible in male 9.9-14.7 mm. Palpi elongate, slender, terminal segment securiform. Antennae elongate, antennomeres 1-3 and the base of 4 with small white reclinate setae dorsally; antennomeres 5-11 with brown pubescence. Eyes small, moderately convex. Frons markedly impressed, with fine scattered round punctures and an irregular median tubercle. Vertex smooth, with small scattered round punctures.

Pronotum cordiform, with broad lateral flanges, distinctly broader than head in both sexes. Two well-defined round or oval patches of short reclinate yellow setae present, one patch on each of the lateral flanges of the pronotum. Pronotum in male with large longitudinal median impression and with two large basal flanges projecting over base of elytra, lateral margins of flanges markedly elevated, apical margins oblique. Pronotum in female markedly impressed medially, lacking basal flanges but with two large, broad tubercles at base. Pronotal surface rugosely punctate medially, smooth with scattered small round punctures otherwise. Scutellum triangular, small and nearly obsolete. Elytra ovate, moderately convex. Elytral surface smooth, with 8 linear striate interneurs (feebly impressed or nearly obsolete in South African specimens) and scattered small round punctures. Elytral disc with short, scattered brown setae. Lateral margins of elytra with a well-defined band of short white reclinate setae. Femora large, massive, with large round punctures. Tibiae elongate, slender, with lateral carinae, protibiae with antennal cleaner notch and a single stout subtending seta, meso-and meta-tibiae thickened at end, with dense reclinate brown setae towards apices and an apical setal fringe, tibial spurs 1-2-2. Tarsi stout, densely setose, protarsi in male broadly expanded, with comb-like setae ventrally.

Abdomen convex, shining, with numerous small round punctures and transverse wrinkles, especially towards lateral margin of ventrites. Apex of sternum VII feebly emarginate in male and broadly rounded in female. Male aedeagus stout, thick (Figure 28).

**Figures 3–11.**
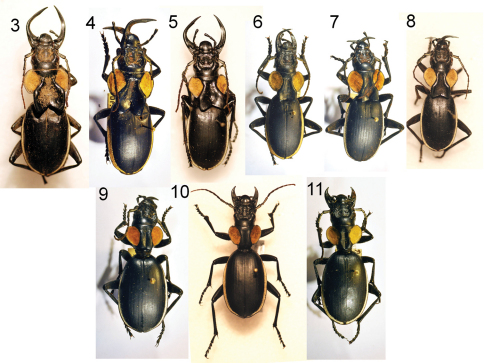
Six adult males **(3–8)** and three adult females **(9–11)** of *Anthia thoracica* (Thunberg), showing variation in male mandible length, in the size of the pronotal flanges in males, and in body size in both sexes. **3** male, Willowmore, Eastern Cape Province, RSA, NMNH **4** male, Lichtenburg, North West Province, RSA, TMSA **5** male, Queenstown, Eastern Cape Province, RSA, NMNH **6** male, Thabina, Gauteng Province, RSA, TMSA **7** male, Bushbuckridge, Mpumalanga Province, RSA, TMSA **8** male, Lichtenburg, North West Province, RSA, NMNH **9** female, Farm Alfa, Mpumalanga Province, RSA, TMSA **10** female, vic. Hazyview, Mpumalanga Province, RSA, NMNH **11** female, Bothaville, Free State Province, RSA, TMSA.

#### Variation.

Males exhibit considerable variation in the size and length of mandibles and in the size of the basal flange on the pronotum (Figures 3-8). Females also exhibit some variability in overall body size (Figures 9-11).

#### Adult activity patterns.

Unimodal, with greatest activity from October to March (Figure 36).

#### Material Examined.

164 pinned adult specimens from the following localities: Republic of South Africa: Eastern Cape Province: Algoa Bay, Despatch, Grahamstown, Port Elizabeth, Port St. Johns, Queenstown, Willowmore. Free State Province: Bothaville, Hendrik Verwoerd Dam, Krugersdrift Dam, Vanwyksfontein Farm, Winburg. Gauteng Province: Boksburg, Cullinan, Florida, Heidelberg, Johannesburg, Pienaars River, Pretoria, Thabina, Valhalla, Zoutpan Pta. KwaZulu-Natal Province: Hluhluwe, Ndumu, Pongola River, “E. Zululand,” “Zululand.” Limpopo Province: Groblersdal, Leydsdorp, Messina, Mogaladi, Mokeetse, Pietersberg, 20-26 miles NE of Pietersberg, Pumbe Sands, Shilouvane, Shingwedzi, Warm Baths, Zebediela, Zoutpansberg. Mpumalanga Province: Barberton, Bushbuckridge, Farm Alfa, Elands River/Middelburg, Groot draai on the Olifants River, Hazyview, vic. Hazyview, Malelane, Nelspruit, Numbi Gate, N’waswitshaka Research Camp, Skukuza, Stolsnek,Waterval pass, Waterval river pass. Northern Cape Province: De Aar, Kimberley. North West Province: Hartebeespoort Dam, Lichtenburg, Mafeking, Rustenburg, 14 miles E Ventersdorp. Western Cape Province: Cape of Good Hope, Cape Town, Dendron. [Additional material was examined from Botswana, Mozambique, Namibia, Tanzania, and Zimbabwe.]

#### Notes on Taxonomy.

*Carabus thoracicus* and *Carabus fimbriatus* are the names given by Thunberg in the same paper to male and female specimens of the present species, a fact which was first noted by Dejean (1825). The name *Carabus thoracicus* has page priority and was selected by Dejean (1825) as the valid name for the species. Dohrn (1882) described a form of this species with slender elytra from South Africa under the name *Anthia portentosa*. Because the name *Anthia portentosa* was already occupied, Rousseau (1905) in the Genera Insectorum proposed the replacement name *Anthia dohrni*. However, no replacement name is needed, as individuals with slender elytra occur throughout the range of the species and thus *Anthia portentosa* Dohrn should simply be treated as a synonym of *Anthia thoracica*. The name *Anthia thoracica* var. *stigmodera* was a manuscript name of Chaudoir’s which Péringuey published in 1896; it refers to a form of this species in which the elytral interneurs are more markedly impressed.

### Anthia maxillosa

(Fabricius, 1781)

http://species-id.net/wiki/Anthia_maxillosa

**Figures 12–17.**
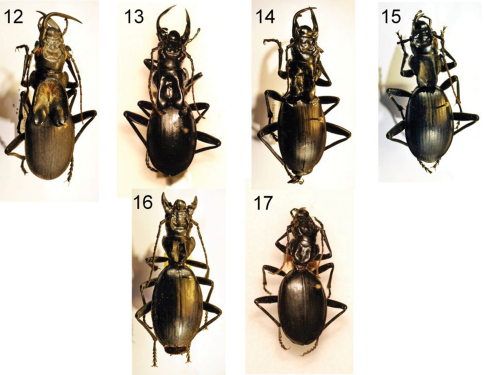
Four adult males **(7–15)** and two adult females **(16–17)** of *Anthia maxillosa* (Fabricius), showing variation in male mandible length, in the size of the pronotal flanges in males, and in body size in both sexes. **12** male, Reichsfontein Gate, Richtersveld National Park, Northern Cape Province, RSA, TMSA **13** male, Free State Province, RSA, NMNH **14** male, Namaqualand, Waterval Farm, Northern Cape Province, RSA, TMSA **15** male, Calvinia, Northern Cape Province, RSA, TMSA **16** female, Willowmore, Eastern Cape Province, RSA, TMSA **17** female, Grootmist, North West Province, RSA, NMNH.

Figures 12–17 29 33 37

Carabus maxillosus Fabricius (1781:298).
Anthia atra Chaudoir (1843:717); synonymized by Péringuey (1896:372).
Anthia fabricii Crotch (1871:3) (unnecessary replacement name).

#### Type Locality.

“Cap. bon. sp.” (= Cape of Good Hope).

#### Type Depository.

*Carabus maxillosus*, Zoological Museum of the University of Copenhagen; *Anthia atra*, Muséum National d’Histoire Naturelle, Paris.

#### Diagnosis.

Easily separated from sympatric species of *Anthia* by the lack of patterned setae on the pronotum and elytra. Scattered white setae may be present along the elytral margins in unrubbed specimens, but these do not form the distinct bands that are found in the other South African *Anthia* species.

#### Description.

Body size massive, length of male 42.0-45.0 mm (exclusive of mandibles), length of female 40.5-45.8 mm. Integument black.

Head elongate, prognathous. Mandibles sexually dimorphic and as described for *Anthia thoracica* except that the left mandible of the male is more markedly recurved. Length of right mandible in male 9.3-12.6 mm. Palpi as in *Anthia thoracica* except terminal maxillary palpomere more markedly securiform. Antennae as described for *Anthia thoracica*, including vestiture. Eyes, frons, and vertex as described for *Anthia thoracica*.

Pronotum cordiform, lateral flanges present but not as broadly expanded as in *Anthia thoracica*, pronotum still broader than head in both sexes. Form of pronotal base is sexually dimorphic as in *Anthia thoracica*, with the apical margins of the flanges in male oblique or slightly curved. Pronotum lacking dorsal setae, surface smooth and shining, with scattered small round punctures. Scutellum as in *Anthia thoracica*. Elytra ovate, markedly convex. Elytral surface sculpture as in *Anthia thoracica*; vestiture in unrubbed specimens composed of scattered brown setae dorsally and a few scattered white setae laterally, never forming well-defined bands. Apex of elytra rounded in females, slightly more pointed in males. Femora and tibiae as in *Anthia thoracica* except with scattered stout black setae. Tarsi as described for *Anthia thoracica* including sexual dimorphism.

Abdomen as in *Anthia thoracica* except abdominal sterna not as markedly wrinkled laterally. Abdominal sternum VII broadly emarginate at apex in male, broadly rounded at apex in female. Male aedeagus elongate, slender (Figure 29).

#### Variation.

Males exhibit considerable variation in the size and length of mandibles and in the size of the basal flange on the pronotum (Figures 7-15). Females also exhibit some variability in overall body size (Figures 16-17).

#### Adult activity patterns.

Unimodal, with greatest activity August-October (Figure 37).

#### Material Examined.

235 pinned adult specimens from the following localities: Republic of South Africa: Eastern Cape Province: 20 miles S Aberdeen, Aberdeen-Beaufort West, Despatch, Grahamstown, Willowmore. Free State Province: Bothaville, no locality specified. Limpopo Province: Grootdraai, Zoutpansberg. Mpumalanga Province: Barberton, Lydenburg. Northern Cape Province: Calvinia, 30 km W Calvinia, De Aar, Duineveld near Stampriet, Kenhardt, Marydale, Nieuwoudtville, Nossob Camp in Kalahari Park, Pofadder, Strydenburg, Van Rhyn’s Pass, Victoria West. Namaqualand region [in Northern Cape]: Braakrivier Mouth, Dikdoorn Farm, Gemsbokvlakte Farm, Harslagkop, Hoekbaai, Katdoringvlei, Klein Kogel Fontein, Kotzesrus, Nababiep, Oograbies, 36 miles E Port Nolloth, Port Nolloth, Quaggasfontein, Rietport Farm, Rooidam Farm, 9 miles S Springbok, 18 km S Springbok, 50 km E Springbok, Springbok-Mesklip, Stallberg Valley, Stinkfontein, 3 km NW Titiesbagi, Vogelklip, Waterval Farm, Wildpaarde Hoek. Richtersveld region [in Northern Cape]: Brakfontein, Buffelsriver valley, Helskloof, Holgat Mouth, 10 W Kuboos, Manganese Mine, Reichsfontein Gate in Richtersveld National Park. North West Province: Grootmist, Haartebeespoort Dam. Western Cape Province: Cape Town, Cedarberg, Koekenaap, Kookfontein, Longkloof, Matiesfontein, Skulpbaai, Touws River, Vanwyksfontein, Zwartskraal farm. [Additional material was examined from Botswana, Namibia, and Zimbabwe.]

#### Notes on Taxonomy.

There has been considerable confusion in the literature and in collections regarding the identity of this species. Most of the confusion is the result of various authors mistakenly associating the name *Carabus maxillosus* Fabricius (1781:298) with the name *Manticora maxillosa* Fabricius (1781:320). These two names were proposed in separate genera and the identities of the taxa to which these names refer are quite clear from the original descriptions. *Carabus maxillosus* is said to have glabrous elytra and two projecting “lamellae” on the base of the thorax; the term “lamellae” accurately describes the modified basal flanges of the pronotum in males of this species, a feature which places this taxon into the modern carabid genus *Anthia*. In contrast, *Manticora maxillosa* is said to have mandibles with a basal tooth and elytra with serrate lateral margins and small tubercles on the disc, features which are not found in *Anthia* but which are commonly encountered in the modern-day sympatric cicindeline genus *Manticora* F. These two names appear in other, subsequent works by Fabricius but there is always a clear distinction between *Carabus maxillosus* with its basal pronotal flanges (Fabricius 1787:194; Fabricius 1801:220, as *Anthia maxillosa* following Weber 1801:17) and *Manticora maxillosa* with its scabrous elytra (Fabricius 1787:220; Fabricius 1801:167). Crotch (1871) erroneously considered *Carabus maxillosus* to be a junior homonym of *Manticora maxillosa*, and proposed the replacement name *Anthia fabricii* for the anthiine species. This replacement name was subsequently adopted by Csiki (1929) in the Coleopterorum Catalogus and consequently is widely used in collections. It is, however, entirely unnecessary, as the two names refer to different taxa and were originally proposed in different genera.

Csiki (1929) listed 18 taxa described from southern and eastern Africa as synonyms of *Anthia maxillosa* (which he called *Anthia fabricii*). These names all need to be carefully reviewed in order to determine whether they represent valid species.

### Anthia cinctipennis

Lequien, 1832

http://species-id.net/wiki/Anthia_cinctipennis

**Figures 18–27.**
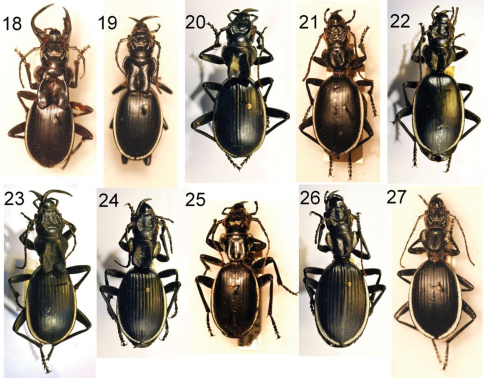
Three adult males **(18–20)** and two adult females **(21–22)** of *Anthia cinctipennis* Lequien, showing variation in male mandible length, in the size of the pronotal flanges in males, and in body size in both sexes. **18** male, Cullinan, Gauteng Province, RSA, NMNH **19** male, Cradock, Eastern Cape Province, RSA, NMNH **20** male, Nauwkluft, Namibia, TMSA **21** female, Grahamstown, Eastern Cape Province, RSA, NMNH **22** female, Langjan Nature Reserve, Limpopo Province, RSA, TMSA. Three adult male **(23–25)** and two adult females **(26–27)** of *Anthia circumscripta* Klug, showing variation in male mandible length, in the size of the pronotal flanges in males, and in body size in both sexes. **23** male, Rehoboth, Namibia, TMSA **24**, male, Namib Desert, Namibia, TMSA **25** male, Zimbabwe, NMNH **26** Namib Desert, Namibia, TMSA **27** Ganab, Namibia, NMNH.

Figures 18–22 30 34 38

Anthia cinctipennis Lequien (1832:unpaginated).
Anthia hottentota Olliff (1889:368-369), synonymized by Csiki (1929:378).
Anthia limbipennis Chaudoir (1861:567), synonymized by Csiki (1929:378).
Anthia pachyoma Chaudoir (1883:26), synonymized by Csiki (1929:378)

#### Type Locality.

“Cap de Bonne-Espérance” (= Cape of Good Hope).

#### Type Depository.

*Anthia cinctipennis*, *Anthia limbipennis*, and *Anthia pachyoma*, Muséum National d’Histoire Naturelle, Paris; *Anthia hottentota*, Hope Department of Entomology, University Museum, Oxford University.

#### Diagnosis.

Easily separated from *Anthia thoracica* by the lack of large round or ovate setal patches on the pronotum, and easily separated from *Anthia maxillosa* by the presence of a band of white setae along the lateral margins of the elytra. Less easily separated from *Anthia circumscripta*, although unrubbed specimens of the latter species always have scattered white setae on the lateral flanges of the pronotum. Male genitalia of *Anthia circumscripta* and *Anthia cinctipennis* are also diagnostic, with the aedeagus slender in *Anthia cinctipennis* and stouter and more robust in *Anthia circumscripta* (Figures 30, 21). Judging by the number of museum specimens examined, *Anthia cinctipennis* is also much more frequently encountered in RSA than *Anthia circumscripta*, which is known from relatively few specimens from RSA.

#### Description.

Body size massive, length of male 41.3–43.8 mm (exclusive of mandibles), length of female 43.5-48.8 mm. Integument black.

Head elongate, prognathous. Mandibles sexually dimorphic and as described for *Anthia maxillosa* except that right mandible of the male has a broad tooth along inner margin. Length of right mandible in male 8.9-10.7 mm. Palpi as in *Anthia maxillosa*. Antennae as described for *Anthia thoracica*, including vestiture. Eyes, frons, and vertex as described for *Anthia thoracica*.

Pronotum as in *Anthia maxillosa*, no dorsal setae present. Basal flange of pronotum well-developed in males; apex of this flange oblique or slightly curved. Pronotal surface markedly shining, with scattered small round punctures. Scutellum as in *Anthia thoracica*. Elytra broad, ovate and somewhat more flattened than in other sympatric *Anthia* species. Elytral sculpture and vestiture as in *Anthia thoracica*. Femora, tibiae, and tarsi as in *Anthia maxillosa*. Mesotibiae distinctly broadened at apex in both sexes, with a patch of short reddish setae on expanded portion.

Abdomen as in *Anthia maxillosa*. Abdominal sternum VII rounded but with a small shallow notch at tip in male, broadly rounded in female. Male aedeagus elongate, slender (Figure 30).

#### Variation.

Males exhibit considerable variation in the size and length of mandibles and in the size of the basal flange on the pronotum (Figures 18-20). Females also exhibit some variability in overall body size (Figure 21-22).

#### Adult activity patterns 

Bimodal (Figure 38), with an activity peak from September-January in central and northeastern RSA (Free State, Gauteng, Limpopo, and Mpumalanga Provinces) and another, smaller activity peak in July in the Kalahari Gemsbok Park and other areas in Northern Cape Province.

#### Material Examined.

131 pinned adult specimens from the following localities: Republic of South Africa: Eastern Cape Province: Cradock, Graaff Reinet, Grahamstown, 14 miles E Middelburg. Free State Province: Bothaville, Reddersburg, Smithfield. Gauteng Province: Bronkhorstspruit, Cullinan, Johannesburg, Pretoria, Rhenosterpoort, Valhalla, Zoutpan Pta. KwaZulu-Natal Province: Elandskraal, “E. Zululand.” Limpopo Province: Dzombo Plots, Gravelotte, Grootdraai, Langjan Nature Reserve, Louis Trichardt, Messina, Nyandu Sandveld, Nylsvley, Penge, 20-26 km NE of Pietersburg, Pietersburg, Shilouvane, Warm Baths, Woodbush, Zoutpansberg. Mpumalanga Province: Barberton, Loskop, Lydenburg, Pilgrim’s Rest, Satara, Skukuza, Waterval, Waterval Pass. Northern Cape Province: Kimberley, Marydale, Richmond, 47 miles N of van Rhynsdorp, Kalahari Gemsbok Park: Farm Mara, 25 km S of Mata Mata, Mata Mata, Twee Rivieren. [Additional material was examined from Botswana, Mozambique, Tanzania, and Zimbabwe.]

### Anthia circumscripta

Klug, 1853

http://species-id.net/wiki/Anthia_circumscripta

Figures 23–27, 31 35 39

Anthia circumscripta Klug (1853:245).

#### Type Locality.

“Tette” (= Tete, Mozambique).

#### Type Depository.

*Anthia circumscripta*, Museum für Naturkunde, Humboldt-Universität, Berlin.

#### Diagnosis.

Unrubbed specimens have scattered areas of white setae on the lateral flanges of the pronotum, which is a diagnostic feature for this species. Rubbed specimens resemble *Anthia cinctipennis* but males can be separated by the structure of the male genitalia which are slender in *Anthia cinctipennis* and stouter and more robust in *Anthia circumscripta* (Figures 30, 31). This species is known from relatively few specimens from RSA; however, it is represented in museum collections by large series from Botswana and Namibia, where it is evidently more frequently collected.

#### Description.

Body size massive, length of male 41.3-48.0 mm (exclusive of mandibles), length of female 42.6-50.4 mm.

Head elongate, prognathous. Mandibles larger in males than in females. Length of right mandible in male 7.4-8.6 mm. Palpi as in *Anthia maxillosa*. Antennae, eyes, frons, and vertex as in *Anthia thoracica*.

Pronotum cordiform, shape as in *Anthia cinctipennis* except that the basal flanges in male are often much smaller; apical margin of flanges transverse, with distinct emargination at apex of both flanges. Lateral flanges of pronotum with a more-or-less distinct patch of scattered short white reclinate setae. Pronotal surface sculpture smooth and shining, markedly punctate with large round punctures. Scutellum as in *Anthia thoracica*. Elytra elliptical-oval, convex medially. Vestiture as in *Anthia thoracica*. Each elytron with 8 distinct longitudinal striate interneurs, which may be feebly to somewhat markedly impressed; each interneur with a row of small round punctures, remainder of surface with scattered small round punctures. Legs as in *Anthia maxillosa*, mesotibiae modified in both sexes as described for *Anthia cinctipennis*.

Abdomen as in *Anthia thoracica*. Male aedeagus stout, robust (Figure 31).

**Figures 28–31.**
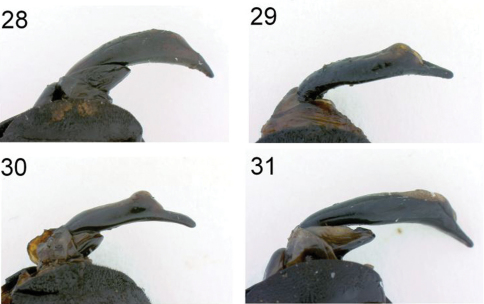
Dorsal view of abdominal apex, with aedeagus extended **28** *Anthia thoracica* (Thunberg) **29** *Anthia maxillosa* (Fabricius) **30** *Anthia cinctipennis* Lequien **31** *Anthia circumscripta* Klug.

#### Variation.

Males exhibit considerable variation in the size and length of mandibles and in the size of the basal flange on the pronotum (Figures 23-25). Females also exhibit some variability in overall body size (Figures 26-27).

#### Adult activity patterns.

Distinctly bimodal (Figure 39), with populations from the Namib and Kalahari deserts having a July activity peak while populations from eastern Botswana, Zimbabwe and the few RSA records have an activity peak in November-March. Figure 39 shows records from throughout southern Africa, since only a very few specimens from RSA were available for study.

#### Material Examined.

2 pinned adult specimens from the following localities: Republic of South Africa: Free State Province: Golden Gate. KwaZulu-Natal Province: Maritzburg. [Additional material was examined from Botswana, Kenya, Namibia, Tanzania, Zambia, and Zimbabwe].

### Taxonomic note on *Apristus promontorii* Péringuey

Obst (1901) erroneously included an “*Anthia promontorii* Péringuey (1898)” in his list of species of the genus *Anthia*, with a comment that he had not actually examined the original description of the species. Had Obst consulted Péringuey’s original description (Péringuey 1898:329), it would have been readily apparent that the species in question was described in the genus *Apristus* Chaudoir (Coleoptera: Carabidae: Lebiini) rather than *Anthia* Weber. Furthermore the species could not possibly belong to the tribe Anthiini, as its maximum dimensions were given as 3.5 mm in length and 1.5 mm in width, measurements that are far smaller than those of any known species of Anthiini. Following Obst, Csiki (1929:381) listed *Anthia promontorii* Péringuey as a species *incertae sedis* under the genus *Anthia* in the Coleopterorum Catalogus. This placement has been followed by other authors and cataloguers, although clearly incorrect. The name *Anthia promontorii* should be treated as a synonym of *Apristus promontorii*.

### Comparative distribution of *Anthia* species in the Republic of South Africa

**Figure 32.**
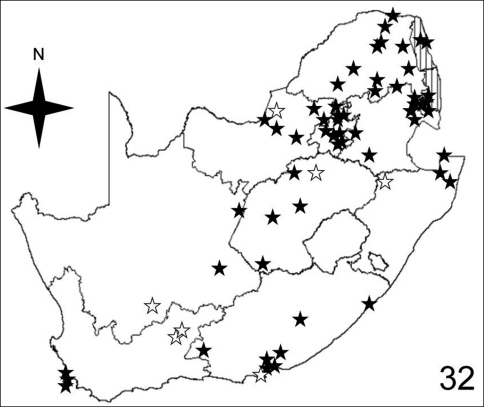
Distribution map showing collecting localities of museum specimens of *Anthia thoracica* (Thunberg) in the Republic of South Africa. Dark stars indicate specimens that we personally examined; white stars indicate literature records.

**Figure 33.**
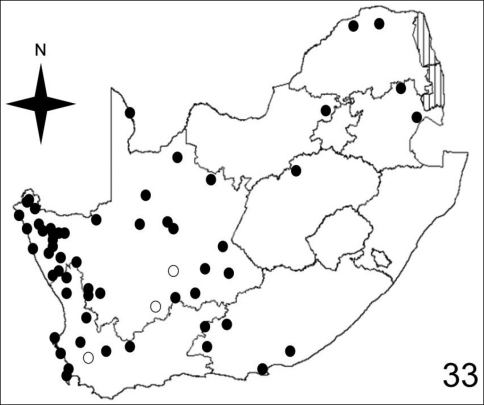
Distribution map showing collecting localities of museum specimens of *Anthia maxillosa* (Fabricius) in the Republic of South Africa. Dark circles indicate specimens that we personally examined; white circles indicate literature records.

**Figure 34.**
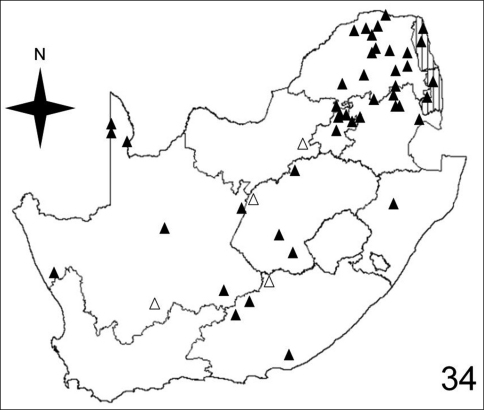
Distribution map showing collecting localities of museum specimens of *Anthia cinctipennis* Lequien in the Republic of South Africa. Dark triangles indicate specimens that we personally examined; white triangles indicate literature records.

**Figure 35.**
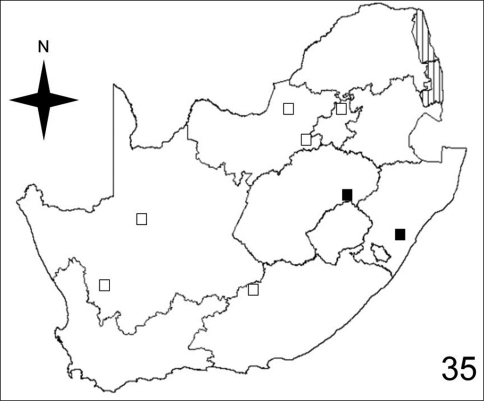
Distribution map showing collecting localities of museum specimens of *Anthia circumscripta* Klug in the Republic of South Africa. Dark squares indicate specimens that we personally examined; white squares indicate literature records.

Figures 32–35 illustrate the distribution of species of *Anthia* in the Republic of South Africa based on museum specimen records, literature records, and our own recent collections. The individual species show markedly different patterns of distribution. *Anthia thoracica* has been most frequently collected in the northern and eastern portions of the country (Figure 32), particularly in Gauteng, Limpopo, and Mpumalanga Provinces. *Anthia maxillosa* has been most frequently collected in the west (Figure 33), particularly the Western Cape and Northern Cape Provinces. *Anthia cinctipennis* has a distribution pattern somewhat similar to that of *Anthia thoracica* (Figure 34), with a high concentration of records in Gauteng, Limpopo, and Mpumalanga Provinces, while *Anthia circumscripta* appears to be rare everywhere in the Republic of South Africa (Figure 35), with very few literature or specimen records. As noted above, *Anthia circumscripta* has been collected in much greater numbers in Botswana and Namibia, and forms a characteristic part of the *Anthia* fauna in both the Kalahari and Namib deserts.

### Association of *Anthia* species with terrestrial ecoregions and veld types in the Republic of South Africa

Schmidt (2001, 2002) and Schmidt and Gruschwitz (2002) were the first to note that species of Anthiini appear to be closely associated with particular vegetation communities in southern Africa. We extend this analysis here using a new map and classification of terrestrial ecoregions that have been developed for continental Africa and Madagascar by Burgess et al. (2004). Comparisons between the map presented by Burgess et al. (2004) and the species distribution maps presented in this paper (Figures 32-35) clearly show the associations between the distributions of individual species of *Anthia* and the terrestrial ecoregions recognized by Burgess et al. (2004) in southern Africa. To examine the association of *Anthia* species with vegetation communities, we also compared our *Anthia* distribution maps against an older but nonetheless widely-used vegetation map which shows the distribution of various veld types in South Africa (Acocks 1988). For this comparison, we used Acocks’ Map 2, which is intended to show actual (as opposed to historic or potential future) vegetation communities in the Republic of South Africa.

Adults of species of *Anthia* are widely distributed throughout the Republic of South Africa (Figures 32–35) and have been collected in twelve of the seventeen terrestrial ecoregions recognized by Burgess et al. (2004). The five ecoregions with no records of *Anthia* species are: Kalahari *Acacia* Woodlands; KwaZulu-Cape Coastal Forest Mosaic; Knysna-Amatole Montane Forests; Montane Fynbos and Renosterveld; and Southern African Mangroves. In some cases (e.g. Southern African Mangroves) this absence of records may represent a true absence of *Anthia* beetles from the ecoregion, while in other cases (e.g. Kalahari *Acacia* Woodlands) the absence of records is likely due to the relatively small amount of this particular ecoregion that occurs in the Republic of South Africa and the absence of surveys for *Anthia* species in those particular small habitat patches.

Adults of *Anthia thoracica* have been collected in eight of the seventeen terrestrial ecoregions recognized by Burgess et al. (2004) in the Republic of South Africa: Highveld grasslands (36.1% of collecting sites); Zambezian and Mopane Woodlands (19.7% of collecting sites); Southern Africa bushveld (13.1% of collecting sites); Nama Karoo (11.5% of collecting sites); Albany Thickets (8.2% of collecting sites); Maputaland Coastal Forest Mosaic (4.9% of collecting sites); Lowland Fynbos and Renosterveld (4.9% of collecting sites); and Drakensburg Alti-Montane Grasslands and Woodlands (1.6% of collecting sites).

Adults of *Anthia maxillosa* have been collected in eight of the seventeen terrestrial ecoregions recognized by Burgess et al. (2004) in the Republic of South Africa: Succulent Karoo (48.3% of collecting sites); Nama Karoo (25.9% of collecting sites); Lowland Fynbos and Renosterveld (6.9% of collecting sites); Kalahari Xeric Savanna (5.2% of collecting sites); Highveld Grasslands (5.2% of collecting sites); Albany Thickets (3.4% of collecting sites); Southern Africa Bushveld (3.4% of collecting sites); and Drakensburg Montane Grasslands, Woodlands, and Forests (1.7% of collecting sites).

Adults of *Anthia cinctipennis* have been collected in eight of the seventeen terrestrial ecoregions recognized by Burgess et al. (2004) in the Republic of South Africa: Highveld grasslands (36.2% of collecting sites); Southern Africa Bushveld (25.5% of collecting sites); Nama Karoo (14.9% of collecting sites); Zambezian and Mopane Woodlands (10.6% of collecting sites); Kalahari Xeric Savanna (6.4% of collecting sites); Succulent Karoo (2.1% of collecting sites); Maputaland-Pondoland Bushland and Thickets (2.1% of collecting sites); and Drakensburg Montane Grasslands, Woodlands, and Forests (2.1% of collecting sites).

Adults of *Anthia circumscripta* have been collected in three of the seventeen terrestrial ecoregions recognized by Burgess et al. (2004) in the Republic of South Africa: Nama Karoo (37.5% of collecting sites); Highveld Grasslands (37.5% of collecting sites); and Drakensburg Montane Grasslands, Woodlands, and Forests (25% of collecting sites). In adjacent countries (Namibia, Botswana, Zimbabwe), this species has also been collected in the Namib Desert, Kalahari Xeric Savanna, and Southern Africa Bushveld ecosystems.

To compare these findings across the four *Anthia* species and the seventeen terrestrial ecoregions in the Republic of South Africa, we used the Spearman rank correlation coefficient (Lowry 2011). We use the Spearman rank correlation coefficient rather than standard regression because we do not know whether the numbers of collecting sites are distributed normally across the set of ecoregions (Lowry 2011). The Spearman rank correlation coefficient tests the significance of correlation between two lists of values. A test statistic close to 1 or -1 indicates that the relative rankings expressed in the two lists of values are markedly correlated. To obtain lists of values for each of the four species of *Anthia*, we calculated the percentage of the total number of collecting sites in each of the seventeen terrestrial ecoregions listed by Burgess et al. (2004) for the Republic of South Africa (Table 1). Next, the Spearman rank correlation coefficient was calculated for each of the possible comparisons of the lists of values in each of the species columns in Table 1, using an online calculator developed by Wessa (2011). These coefficients are reported in Table 2. Inspection of Table 2 shows clearly that for all four species, the relative percentages of collecting sites in each ecoregion are not significantly similar at the 95% level (test statistic between .95 and 1.0 or between -.95 and -1.0). From these findings, it can be inferred that these four species appear to be distributed across the set of southern African ecoregions in statistically dissimilar ways. These results make intuitive sense, given the obvious visual differences in the species distribution maps for these taxa (Figures 32-35).

**Table 1.** Percentage of collecting sites for each of four species of *Anthia* in each of the seventeen terrestrial ecoregions in the Republic of South Africa.

**Terrestrial Ecoregion**	**% collecting localities for *Anthia thoracica***	**% collecting localities for *Anthia maxillosa***	**% collecting localities for *Anthia cinctipennis***	**% collecting localities for *Anthia circumscripta***
Maputaland Coastal Forest Mosaic	4.9	0.0	0.0	0.0
KwaZulu–Cape Coastal Forest Mosaic	0.0	0.0	0.0	0.0
Knysna-Amatole Montane Forests	0.0	0.0	0.0	0.0
Zambezian and Mopane Woodlands	19.7	0.0	10.6	0.0
Southern Africa Bushveld	13.1	3.4	25.5	0.0
Kalahari Acacia Woodlands	0.0	0.0	0.0	0.0
Highveld Grasslands	36.1	5.2	36.2	37.5
Drakensburg Montane Grasslands, Woodlands, and Forests	0.0	1.7	2.1	25.0
Drakensburg Alti-Montane Grasslands and Woodlands	1.6	0.0	0.0	0.0
Maputaland-Pondoland Bushland and Thickets	0.0	0.0	2.1	0.0
Lowland Fynbos and Renosterveld	4.9	6.9	0.0	0.0
Montane Fynbos and Renosterveld	0.0	0.0	0.0	0.0
Albany Thickets	8.2	3.4	0.0	0.0
Kalahari Xeric Savanna	0.0	5.2	14.9	0.0
Nama Karoo	11.5	25.9	6.4	37.5
Succulent Karoo	0.0	48.3	2.1	0.0
Southern African Mangroves	0.0	0.0	0.0	0.0

**Table 2.** Spearman rank correlation coefficients from pairwise comparisons of the columns of values for each species in Table 1.

	*Anthia thoracica*	*Anthia maxillosa*	*Anthia cinctipennis*
*Anthia maxillosa*	0.319655		
*Anthia cinctipennis*	0.441241	0.50938	
*Anthia circumscripta*	0.347861	0.444142	0.492868

**Table 3.** Percentage of collecting sites for each of four species of *Anthia* in each of the ten veld types in the Republic of South Africa.

**Veld Type**	**% collecting localities for *Anthia thoracica***	**% collecting localities for *Anthia maxillosa***	**% collecting localities for *Anthia cinctipennis***	**% collecting localities for *Anthia circumscripta***
Desert	0.0	14.3	2.1	0.0
Succulent Karoo	0.0	2.4	0.0	0.0
Karoo	11.3	63.1	18.8	37.5
Bushveld	51.6	10.7	45.8	12.5
Scrubby mixed Grassveld	0.0	0.0	0.0	0.0
Sweet Grassveld	9.7	1.2	2.1	0.0
Mixed Grassveld	9.7	0.0	2.1	0.0
Sour Grassveld	9.7	1.2	29.2	50.0
Forest and Scrubforest	0.0	0.0	0.0	0.0
Fynbos	8.1	7.1	0.0	0.0

**Table 4.** Spearman rank correlation coefficients from pairwise comparisons of the columns of values for each species in Table 3.

	*Anthia thoracica*	*Anthia maxillosa*	*Anthia cinctipennis*
*Anthia maxillosa*	0.347412		
*Anthia cinctipennis*	0.831126	0.431049	
*Anthia circumscripta*	0.693726	0.401331	0.802852

Adults of *Anthia* species are likewise broadly distributed across the various veld types or large-scale vegetation communities recognized by Acocks (1988) in southern Africa, with collection records of species of *Anthia* from eight of the ten veld types recorded for the Republic of South Africa. The two veld types with no records of *Anthia* species are Scrubby mixed Grassveld and Forest and Scrubforest. It is possible that the lack of records of *Anthia* species from these two veld types is due simply to lack of sampling activities in these two communities, both of which are relatively restricted in geographic extent (Acocks 1988).

Adults of *Anthia thoracica* have been collected in six of the ten veld types: Bushveld (51.6% of collecting sites); Karoo (11.3% of collecting sites); Sweet Grassveld (9.7% of collecting sites); Mixed Grassveld (9.7% of collecting sites); Sour Grassveld (9.7% of collecting sites); and Fynbos (8.1% of collecting sites).

Adults of *Anthia maxillosa* have been collected in seven of the ten veld types: Karoo (63.1% of collecting sites); Desert (14.3% of collecting sites); Bushveld (10.7% of collecting sites); Fynbos (7.1% of collecting sites); Succulent Karoo (2.4% of collecting sites); Sweet Grassveld (1.2% of collecting sites); and Sour Grassveld (1.2% of collecting sites).

Adults of *Anthia cinctipennis* have been collected in six of the ten veld types: Bushveld (45.8% of collecting sites); Sour Grassveld (29.2% of collecting sites); Karoo (18.8% of collecting sites); Desert (2.1% of collecting sites); Sweet Grassveld (2.1% of collecting sites); Mixed Grassveld (2.1% of collecting sites).

Adults of *Anthia circumscripta* have been collected in just three of the ten veld types: Sour Grassveld (50%); Karoo (37.5%); and Bushveld (12.5%).

We again applied the Spearman rank correlation coefficient (Lowry 2011) to examine the relative abundance of collecting sites of the four species of *Anthia* across the ten veld types. Table 3 shows the percentage of collecting sites for each species in each of the veld types. Table 4 shows the Spearman rank correlation coefficient for each pairwise comparison of columns in Table 3, calculated using the web calculator developed by Wessa (2011). Here again the pairwise comparisons between columns are not significantly similar at the 95% level. This findings suggests that the four species of *Anthia* are distributed across the veld type of South Africa in statistically dissimilar ways.

### Seasonality of *Anthia* species in the Republic of South Africa

**Figure 36.**
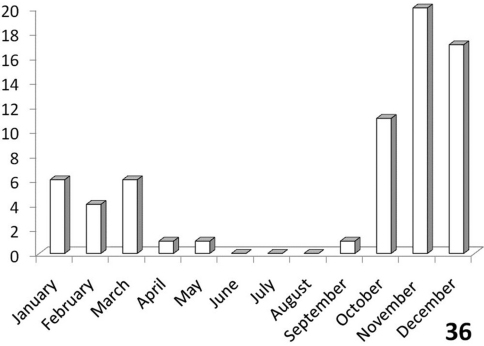
Activity patterns of adults of *Anthia thoracica* (Thunberg) in South Africa, based on museum specimen records and summed by month across all years. The vertical axis indicates numbers of specimens.

**Figure 37.**
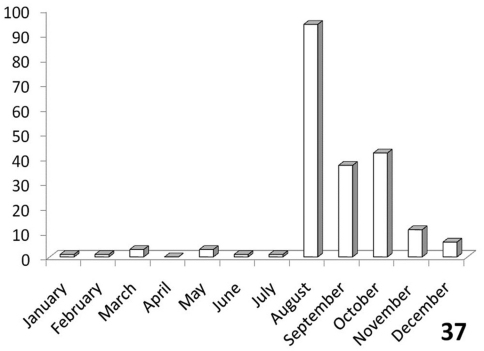
Activity patterns of adults of *Anthia maxillosa* (Fabricius) in South Africa, based on museum specimen records and summed by month across all years. The vertical axis indicates numbers of specimens.

**Figure 38.**
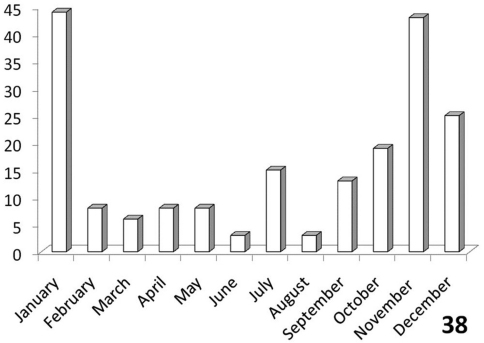
Activity patterns of adults of *Anthia cinctipennis* Lequien in South Africa, based on museum specimen records and summed by month across all years. The vertical axis indicates numbers of specimens.

**Figure 39.**
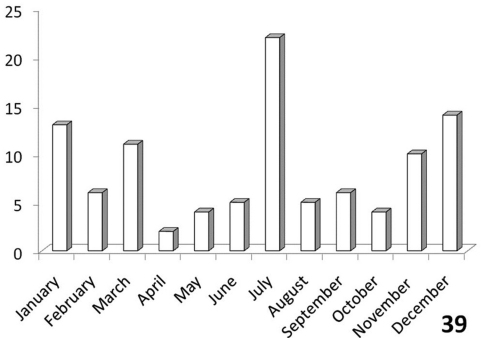
Activity patterns of adults of *Anthia circumscripta* Klug in South Africa, based on museum specimen records and summed by month across all years. The vertical axis indicates numbers of specimens.

The histograms in Figures 36-39 summarize dates of collection of museum specimens of *Anthia* species across all years. These charts suggest that individual species of the genus *Anthia* exhibit marked seasonality in the timing of adult emergence and in the timing of adult activity patterns, as noted previously by Schmidt (2001) and Schmidt and Gruschwitz (2002). In general, emergence and activity patterns in adults of Anthiini appear to be coordinated with the onset of seasonal rains (Schmidt 2001; Schmidt and Gruschwitz 2002). As shown in Figure 36, adults of *Anthia thoracica* were most frequently collected between October and March, corresponding to the seasonal monsoonal rains in the eastern portion of the country (du Toit et al. 2003), where *Anthia thoracica* has been most frequently collected (Figure 32). In contrast, most collections of *Anthia maxillosa* have occurred between August and October (Figure 37), corresponding to the period of winter rains in the Northern and Western Cape provinces (Mares 2002) where this species has been most frequently collected (Figure 33). *Anthia cinctipennis* and *Anthia circumscripta* exhibit similar bimodal activity patterns in the Republic of South Africa (Figures 38 and 39), with populations in the eastern portion of the country active during the summer monsoon rains (September-January and November-March, respectively; Figures 38 and 39), while Kalahari Desert populations of both species are active during July.

### Investigations of possible allometry in secondary sexual characteristics of males of *Anthia* species

Males of species of *Anthia* in the Republic of South Africa often have enlarged and elongated mandibles as well as distinctive flanges on the base of the pronotum (Figures 3–8, 12–15, 18–20, 23–25). Both mandibles and flanges come in large, small, and intermediate sizes, and in general, larger-bodied males appear to have larger mandibles and pronotal flanges than smaller males. We were interested in determining whether mandible and pronotal flange size of these beetles scale isometrically (mandible and flange size increases at the same rate as body size) or allometrically (mandible and flange size increase at a rate disproportionately greater than body size). We measured three variables, right mandible length, pronotal length (from apex to base of flange), and elytral width (a surrogate measure of body size, measured at the widest point with the elytra fully closed in a natural position) for all male specimens of these four *Anthia* species in the NMNH collection. For *Anthia thoracica*, we observed only a very weak correlation between elytral width and mandible length (R^2 ^= 0.61) and between elytral width and pronotal length (R^2^ = 0.63) using standard linear regression. Both R^2 ^values improved with the use of a polynomial (x^2^) function in the regression, to 0.67 and 0.75, respectively. The improved fit with the polynomial function and the generally low R^2^ values from the standard linear regression suggests that both mandible size and pronotal flange size scale non-linearly with respect to body size in this species. In the other three species, mandible length and pronotal length were very poorly correlated with elytral width (R^2^ values ranging from 0.04 to 0.27), suggesting that mandible and pronotal flange size is not correlated with elytral width.

### South African Anthiini as vectors for *Cyaneolytta* larvae (Coleoptera: Meloidae)

Many large-bodied Carabidae in southern Africa have been documented as serving as vectors for the phoretic larvae of species in the blister beetle genus *Cyaneolytta* Péringuey (Bologna et al. 1990; Di Iulio et al. 2003). In the Republic of South Africa, larvae of these beetles have been collected in association with adults of all four species of *Anthia* as well as several sympatric species of *Termophilum* (Di Iulio et al. 2003). The nature of these phoretic associations remains unclear (Di Iulio et al. 2003) although a number of possible explanations have been advanced by Bologna et al. (1990).

### Observations on the biology and behavior of *Anthia thoracica* (Thunberg) in the Kruger National Park

*Anthia thoracica* is one of two species in the genus *Anthia* recorded from Kruger National Park, a large and well-known conservation area which is located in eastern Limpopo and Mpumalanga Provinces, South Africa (du Toit et al. 2003). Recent fieldwork in the southern portion of this park by the senior author has provided us with opportunities to examine certain aspects of the biology of *Anthia thoracica* under both field and laboratory conditions. Protocols for field surveys for *Anthia thoracica* and other Anthiini, as well as methods for laboratory maintenance of captive Anthiini, are described above under the Materials and Methods section.

Occurrences within Kruger National Park: Specimens of *Anthia thoracica* have been collected at the following localities within Kruger National Park: Malelane, Numbi Gate, Pumbe Sands, Shingwedzi, Skukuza, and Stolsnek. In addition, the senior author and associates have recently encountered this species on sand and gravel roads in the vicinity of the Pretoriuskop and Skukuza rest camps, and in the N’waswitshaka Research Camp at Skukuza.

**Figure 40.**
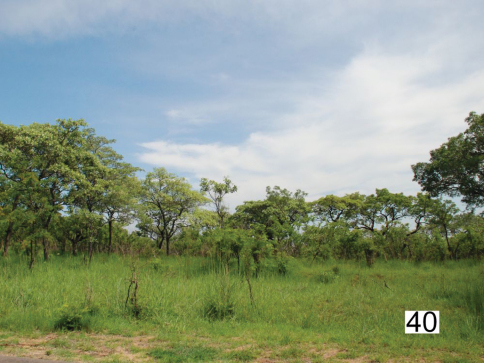
*Terminalia sericea* Burchell woodland near Pretoriuskop, Kruger National Park, habitat for *Anthia thoracica* (Thunberg) and *Termophilum burchelli* (Hope).

**Figure 41.**
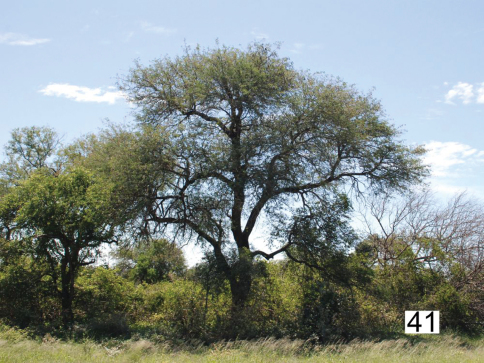
*Acacia nigrescens* Oliver – *Combretum apiculatum* Sonder woodland near Skukuza, Kruger National Park, habitat for *Anthia thoracica* (Thunberg), *Termophilum homoplatum* (Lequien), and *Cypholoba graphipteroides* (Guérin).

Associated ecological communities: Gertenbach (1983) characterized a series of 35 “landscapes” in Kruger National Park which are defined on the basis of plant communities, climate, geology, soils, vegetation, and vertebrate species composition. Adults of *Anthia thoracica* have been collected in the following five landscapes within the Park: Lowveld sourveld bushveld of Pretoriuskop (Gertenbach Landscape 1; Pretoriuskop; Figure 40). Malelane mountain bushveld (Gertenbach Landscape 2; Malelane, Numbi Gate, Stolsnek). Thickets of the Sabie and Crocodile Rivers (Gertenbach Landscape 4; N’waswitshaka Research Camp, Skukuza; Figure 41). *Combretum* spp./*Colophospermum mopane* (Bentham) Leonard (Fabaceae) rugged veld (Gertenbach Landscape 22; Shingwedzi). Pumbe sandveld (Gertenbach Landscape 30; Pumbe Sands). Common geomorphological features in these landscapes include eroding granite, gneiss, or basalt koppies and hills. Extensive sand deposits, sometimes as deep as 6 meters, are also found in many of these landscapes. The silver cluster-leaf tree (*Terminalia sericea* Burchell, Combretaceae; Figure 40) is a major component of the vegetation in several of these landscapes (although less common in the vicinity of Skukuza; Gertenbach 1983).

**Figure 42.**
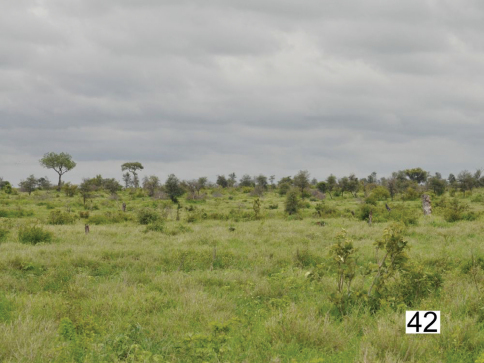
Open grassland savanna near Satara, Kruger National Park, habitat for *Anthia cinctipennis* Lequien, *Termophilum homoplatum* (Lequien), and *Termophilum massilicatum* (Guérin).

Co-occurrence with allied species: We collected adults of *Anthia thoracica* in association with adults of of *Termophilum burchelli* (Hope), *Termophilum homoplatum* (Lequien), *Termophilum massilicatum* (Guérin), and *Cypholoba graphipteroides* (Guérin) (all Carabidae: Anthiini). Adults of *Termophilum homoplatum* and *Termophilum massilicatum* were found primarily in the open savanna areas extending northward area from Skukuza to Satara (Figure 42), while adults of *Termophilum burchelli* and *Cypholoba graphipteroides* were found in denser thickets and woodlands from Pretoriuskop in the south along the banks of the Sabie River to Skukuza (Figures 40-41). The related species *Anthia cinctipennis* Lequien has also been collected in Kruger National Park, with records from Skukuza, Satara, and the Nyandu Sandveld (Figure 42).

Activity period: Specimen records from South Africa (Figure 36) indicate that collections of *Anthia thoracica* have occurred between September and May, with a peak in November-December. Emergence of adults of *Anthia thoracica* appears to be triggered by the onset of seasonal rains (Schmidt 2001), which in Kruger National Park typically occurs in November or December (du Toit et al. 2003). Adult beetles are usually encountered singly, walking rapidly on sand or gravel roads or in open areas of the veld. Our field observations of this species in the Park were conducted during the early rainy season in November and December, corresponding to the time of peak collections of *Anthia* species and other Anthiini as indicated by museum specimen labels. No adults of *Anthia thoracica* were observed during a visit to the Park during the late dry season in September, 2006.

Weather and climate: Adult activity patterns of *Anthia thoracica* appear to be markedly influenced by weather and climatic conditions (Schmidt 2001). As mentioned above, adult emergence is clearly associated with the onset of seasonal rains (Schmidt 2001). In the Kruger National Park, adults can be found diurnally walking on sand and gravel roads and in open areas of the veld immediately following a rainfall event. After a series of days without rain, adult activity patterns change and the foraging adults are only encountered crepuscularly and nocturnally. On subsequent overcast or rainy days, however, adults again become active diurnally. Similar activity patterns with respect to weather and climatic conditions have been noted in populations of the carabid *Tefflus meyerlei delagorguei* in Kruger National Park (Mawdsley et al. 2011).

Defensive behaviors: Adults of *Anthia thoracica* spray copious amounts of highly concentrated formic acid from their pygidial glands when disturbed (Scott et al. 1975; Schmidt 2001). As noted by Huey and Pianka (1977), the spray is often directed towards the head and eyes of the person disturbing the beetle. If picked up by a human, the beetle often directs the spray towards the hands of the person disturbing it. There appears to be a limited supply of acid available to the beetle; the adults of *Anthia thoracica* and *Termophilum* species which we kept in captivity did not spray again after the initial capture. It has been suggested to the authors that the formic acid may be acquired and concentrated by the beetle as a by-product from consuming ants (Hymenoptera: Formicidae). However, other species of Carabidae have the ability to synthesize formic acid in their pygidial glands and to spray this acid as a defensive compound (e.g. Rossini et al. 1997).

Mimicry: *Anthia thoracica* and the sympatric species *Termophilum homoplatum* (Lequien) share similar color patterns which include large round or ovate eyespots, a black, shining dorsal integument, and a narrow white linear band along the outer margin of the elytra (Figures 1, 2). In *Anthia thoracica*, the eyespots are on the pronotum while in *Termophilum homoplatum* the eyespots are on the base of the elytra. Both species occur in similar habitats at the same times of year and exhibit similar fast walking behaviors. Both species are chemically defended, with the capacity to spray similar combinations of highly concentrated formic acid and other acidic compounds from the pygidial glands (Scott, Hepburn, and Crewe 1975). Marshall and Poulton (1902) suggested that the similarity in coloration between these two species may be an example of mimicry. Since both species share a similar noxious defensive behavior, any mimetic interactions would likely be an example of Müllerian mimicry (Wickler 1968). Wickler (1968) emphasizes the importance of identifying potential agents of selection which could be responsible for driving the evolution of mimetic resemblances between species. In Kruger National Park, potential predators which forage for terrestrial arthropods in the areas where Anthiine beetles are encountered and which might serve as agents of selection for mimetic interactions between *Anthia* and *Termophilum* species include chacma baboon (*Papio ursinus* (Kerr)), vervet monkey (*Chlorocebus pygerythrus* Cuvier), banded mongoose (*Mungos mungo* Gmelin), black-backed jackal (*Canis mesomelas* (Schreber)), southern ground hornbill (*Bucorvus leadbeateri* (Vigors)), and the secretary bird (*Sagittarius serpentarius* (Miller)). We observed foraging adults of all of these vertebrate species in the areas that we surveyed for Anthiine beetles, with chacma baboon, vervet monkey, and banded mongoose present in significant numbers (n > 10 individuals) at many sites. The abundance of these potential predators suggests that there may be considerable selection pressures on large carabid beetles that are driving the evolution of chemical defenses, aposematic coloration, and Müllerian mimicry complexes. On balance, the hypothesis of mimicry between these two beetle species seems reasonable.

Prey species: Adults of *Anthia thoracica* in captivity showed few preferences regarding prey items and consumed a wide range of prey species, including representatives of the following orders and families: Coleoptera: Carabidae, Cerambycidae, Curculionidae, Scarabaeidae, Tenebrionidae. Hemiptera: Cicadidae, Cydnidae. Hymenoptera: Formicidae. Isoptera: Termitidae. Lepidoptera: Arctiidae, Geometridae, Noctuidae, Saturniidae. Orthoptera: Acrididae. These insects were captured at lights at night in the N’waswitshaka Research Camp and fed to the live *Anthia thoracica*.

Foraging behavior: Adults of *Anthia thoracica* exhibit a rapid walking behavior which appears to serve multiple functions: foraging for food, detection of mates, and dispersal of adults. In captivity, this behavior was often noticed in adults of *Anthia thoracica* which had not been fed for several hours.

Prey detection: Adults of *Anthia thoracica* were observed to move and vibrate their antennae in the presence of potential prey items, suggesting that chemical cues may form an important part in the detection and recognition of prey items.

Predatory behaviors: Adults of *Anthia thoracica* seized prey using their mandibles and rapidly crushed prey items with the mandibular bases, using the labial and maxillary palpi to hold and manipulate the prey item. Liquid contents of prey and soft tissues were consumed whole while heavily sclerotized parts and appendages (legs, wings) were discarded.

Drinking: Captive adults of *Anthia thoracica* were observed drinking from wet cotton balls which we provided in their containers; in drinking, the adult beetle stands perpendicular to the water source, the ventral surface of the head is pressed against the wet cotton and water is then taken directly into the buccal cavity.

Antennal cleaning: Adults of both sexes of *Anthia thoracica* have an antennal cleaning notch along the inner margin of the protibiae. Adults were observed drawing the antennae through this notch after feeding, after handling by humans, and after being introduced into a new captive holding chamber.

## Concluding Remarks

We hope that the identification materials presented in this paper will be of assistance to entomologists, field biologists, national park managers, and others who want to identify specimens of *Anthia* species in the Republic of South Africa. We also hope that this paper helps to spark additional interest in these large and spectacular members of the South African insect fauna. Species of the genus *Anthia* and related genera are large, common, and conspicuous members of savanna and woodland ecosystems throughout southern and eastern Africa. Our understanding of the natural history of this group is limited at present and further investigations of the life history, immature stages, and biology of these beetles are clearly needed. The genus *Anthia* and its relatives provide excellent opportunities for studying the evolution of chemical defenses, aposematic coloration, and Müllerian mimicry. *Anthia* and its relatives also have attributes which suggest they may be excellent candidates for inclusion in monitoring programs that track ecosystem condition or ecological integrity in southern Africa. Adult activity patterns of *Anthia* species closely track a variety of environmental and climatological variables (Schmidt 2001), and the presence of adult beetles could be taken as indicative of the presence of certain favorable climatic conditions (Schmidt 2001). Adults (and presumably larvae) of *Anthia* species and relatives are voracious predators (Schmidt 2001) and thus the presence of the adult beetles is indicative of a suitable arthropod prey base for both larval and adult development. Our results and those of Schmidt and Gruschwitz (2002) suggest that individual species of *Anthia* show close associations with particular ecoregions or vegetation communities, and thus species of these beetles could potentially also serve as indicators of community condition. The survey protocols for adults of *Anthia* and related genera are relatively straightforward (see Methods above) and can be easily replicated using small teams of surveyors. Identification of species in the genus *Anthia* and related genera is based largely on external surface sculpture and setal patterns (Schmidt 2002), which could be mastered by non-specialists. By making these basic identification materials widely accessible, we hope to stimulate further interest in these fascinating members of the southern African carabid fauna.

## Supplementary Material

XML Treatment for Anthia

XML Treatment for Anthia thoracica

XML Treatment for Anthia maxillosa

XML Treatment for Anthia cinctipennis

XML Treatment for Anthia circumscripta
